# Distinct Effects of Carrageenan and High-Fat Consumption on the Mechanisms of Insulin Resistance in Nonobese and Obese Models of Type 2 Diabetes

**DOI:** 10.1155/2019/9582714

**Published:** 2019-04-15

**Authors:** Sumit Bhattacharyya, Leo Feferman, Joanne K. Tobacman

**Affiliations:** Department of Medicine, University of Illinois at Chicago and Jesse Brown VA Medical Center, Chicago, IL 60612, USA

## Abstract

Exposure to low concentration of the common food additive carrageenan (10 mg/L) for only six days led to glucose intolerance and insulin resistance in the C57BL/6J mouse. Longer exposure produced fasting hyperglycemia but with no increase in weight, in contrast to the HFD. Glucose intolerance was attributable to carrageenan-induced inflammation and to increased expression of GRB10. Both HFD and carrageenan increased p(Ser32)-I*κ*B*α* and p(Ser307)-IRS1, and the increases were greater following the combined exposure. The effects of carrageenan were inhibited by the combination of the free radical inhibitor Tempol and BCL10 siRNA, which had no impact on the HFD-mediated increase. In contrast, the PKC inhibitor sotrastaurin blocked the HFD-induced increases, without an effect on the carrageenan-mediated effects. HFD had no impact on the expression of GRB10. Both carrageenan and high fat increased hepatic infiltration by F4/80-positive macrophages. Serum galectin-3 and galectin-3 binding to the insulin receptor increased by carrageenan and by HFD. Tyrosine phosphorylation of the insulin receptor declined following either exposure and was further reduced by their combination. Carrageenan reduced the activity of the enzyme N-acetylgalactosamine-4-sulfatase (ARSB; arylsulfatase B), which was unchanged following HFD. Dietary exposure to both high fat and carrageenan can impair insulin signaling through both similar and distinct mechanisms.

## 1. Introduction

Carrageenans are sulfated polygalactans present in red seaweeds and are very commonly used as additives in processed foods. Carrageenan improves the texture of processed foods by increasing the solubility of a variety of ingredients, particularly milk proteins. Although carrageenan has been widely used in the laboratory to produce inflammation in animal and cell-based models for decades, its inclusion in processed foods, pharmaceuticals, cosmetics, and other commercial products has continued to increase [1]. Previously, we reported that carrageenan induced glucose intolerance and insulin resistance by two distinct mechanisms, including an increase in phospho(Ser307/312)-IRS1, a negative regulator of insulin signaling, and decline in phospho(Tyr)-IRS1, a positive regulator of insulin signaling [2–4]. Carrageenan activates inflammatory pathways leading to NF-*κ*B nuclear translocation by distinct pathways, including a Toll-like receptor (TLR) 4–B-cell CLL-lymphoma (BCL) 10-mediated pathway and a reactive oxygen species- (ROS-) mediated pathway that lead to activation of the IKK (inhibitor of *κ*B kinase) signalosome which interacts with phospho(Ser307/312)-IRS1 [5–10].

The TLR4-mediated pathway of innate immunity is linked to inhibition of insulin signaling, consistent with the effects of carrageenan on glucose intolerance [2, 11–14]. The IKK-*β* component of the IKK signalosome phosphorylates Ser307(mouse)/Ser312(human) of insulin receptor substrate- (IRS-) 1, negatively regulating insulin signaling and linking the inflammatory and insulin signaling pathways [9, 10]. An additional mechanism whereby carrageenan inhibited insulin signaling is by increasing the mRNA expression of growth factor receptor-bound protein (GRB) 10 [4]. GRB10 is an imprinted, SH2 domain-containing, adaptor protein which binds with receptor tyrosine kinases, including the insulin receptor and insulin growth factor- (IGF-) 1R, and interferes with IRS-1/2 activation by interactions with SH2 domain-containing proteins [4, 15–18]. By inhibiting IR/IRS tyrosine phosphorylation, GRB10 acts as a negative regulator of insulin signaling and growth inhibitor. Enhanced expression of GRB10 by carrageenan inhibits the forward activation of insulin signaling, manifested by decline in phospho(S473)-AKT1 and inhibition of glucose uptake.

The HFD also leads to glucose intolerance and insulin resistance, either directly by inflammatory effects of high-fat exposure or by secondary effects related to obesity [19, 20]. Obesity has been associated with insulin resistance mediated by increased expression of TNF-*α* [21, 22]. An increase in plasma free fatty acid levels in a rat model during euglycemic-hyperinsulinemic clamp studies was associated with an increase in active PKC*θ* [23]. In normal volunteers, euglycemic-hyperinsulinemic clamp studies with lipid infusion were associated with increases in diacylglycerol (DAG) and in membrane-associated PKC isoforms in skeletal muscle [24]. Other studies have noted the role of DAG activation of PKC*θ* in lipid-induced insulin resistance in muscle of obese individuals and individuals with type 2 diabetes, due to the effects on IRS-1 serine and tyrosine phosphorylations [25]. The relative contributions of lipotoxicity with production of reactive oxygen species or of obesity as causes of insulin resistance remain to be fully defined [26].

The experiments in this report present distinct effects of inflammation on insulin resistance in the obese vs. the nonobese model and may help to isolate specific effects attributable to excess lipid exposure and the consequent obesity-related vs. inflammatory-related effects. In mouse models of exposure to carrageenan, HFD, or the combination of carrageenan and HFD, the carrageenan exposure was not associated with increased weight, in contrast to the HFD [3], and the combination of carrageenan and HFD did not lead to a greater weight increase than the HFD alone. Glucose tolerance tests demonstrated that the combination of carrageenan and HFD produced an earlier onset of fasting hyperglycemia and increased the area under the GTT glucose response curve than either interventions alone [3]. Serum keratinocyte chemokine (KC), the mouse homolog of interleukin- (IL-) 8, IL-6, MCP-1, and fecal calprotectin were increased by carrageenan but not by HFD. Hepatic glycogen stores were reduced by carrageenan treatment but not by the HFD. The combination of HFD and carrageenan increased the serum total and non-HDL cholesterol in the combined model, but carrageenan alone had no effect [3].

To address the underlying mechanisms by which the HFD-induced obese model of type 2 diabetes interacts with the carrageenan-induced nonobese model to produce the earlier onset of fasting hyperglycemia and more severe glucose intolerance, experiments were performed to further evaluate the interactions and distinct effects of carrageenan and HFD. Experiments were also performed with palmitic acid-treated HepG2 cells to address the impact of these exposures in cell-based studies without the confounding effect of the obese state. These studies provide additional insight into how systemic inflammation, in the absence of obesity, impacts on insulin signaling, independently and in combination with the metabolic consequences of HFD and obesity.

## 2. Research Design and Methods

### 2.1. Cell and Mouse Models of Carrageenan and High-Fat Exposure

HepG2 cells (ATCC^®^ HB-8065; Manassas, VA), a human hepatic adenocarcinoma cell line, were grown under recommended conditions using minimum essential medium with 10% FBS and were maintained at 37°C in a humidified 5% CO_2_ environment with medium exchange every 2 days [2]. Confluent cells in T-25 flasks were harvested by EDTA-trypsin and subcultured in multiwell tissue culture plates. In most of the experiments, cells were exposed to either *λ*-carrageenan (1 mg/liter; Sigma) and 200 *μ*M palmitic acid (Sigma-Aldrich, St. Louis, MO) in medium with serum for 20 h or *λ*-carrageenan (1 mg/liter) and 200 *μ*M palmitic acid in medium without serum for 4 h. Cells were washed with serum-free medium, and fresh serum-free medium with regular human insulin (Humulin U-100; Lilly, Indianapolis, IN; 20 nmol/L) was added for 10 min. In some experiments, cells were also treated with the PKC inhibitor sotrastaurin (Selleckchem, Houston, TX) at a dose of 250 nM × 24 h [27]. BCL10 siRNA and Tempol were used as previously [5].

Eight-week-old male C57BL/6J mice (*n* = 32 in groups of 8) were purchased (Jackson Laboratory, Bar Harbor, ME) and housed in the Veterinary Medicine Unit at the Jesse Brown VA Medical Center (JBVAMC, Chicago, IL). All procedures were approved by the Animal Care Committees of the University of Illinois at Chicago and the JBVAMC in accord with the Institutional Animal Care and Use Committee (IACUC) standards, as previously [3].

### 2.2. Glucose Determinations and Glucose Tolerance Tests

Whole blood samples from a small tail puncture were collected on glucose strips to measure blood glucose levels by glucometer (OneTouch Ultra 2, LifeScan, Milpitas, CA), as previously [2, 3]. Glucose tolerance tests (GTTs) were performed following overnight 15-hour fasts, with measurements at times 0, 15, 30, 60, and 90 minutes following dextrose injection (2 g/kg IP in filtered PBS). Mean glucose values from at least three mice from each group at each time point were compared. GTTs were carried out at the beginning of the study and at the end.

### 2.3. Serum Insulin Measurements

Plasma insulin levels were measured by a dual-monoclonal antibody sandwich ELISA (Alpco, Salem, NH) in blood samples collected in heparinized capillary tubes at 0 and 30 min during the GTT performed at the onset and termination of the study exposures, as previously [1, 3].

### 2.4. Determination of Glucose-Stimulated Insulin Secretion (GSIS) and the Homeostatic Model Assessment (HOMA) of Insulin Resistance

Glucose-stimulated insulin secretion (GSIS) was measured by determining the fasting insulin levels and insulin levels 30 minutes after dextrose injection during the glucose tolerance test (GSIS = insulin (30 min-0 min) mIU/L) [28]. HOMA-IR was calculated by the formula: HOMA-IR = glucose(mg/dL) × insulin (mIU/L)/405 [29]. The HOMA 2.2 calculations were performed using the computer-based calculator to determine steady-state beta cell function (HOMA-%*β*), insulin sensitivity (HOMA-%S), and insulin resistance (IR) [30].

### 2.5. ELISAs for Phospho(Ser32) Inhibitor of NF-*κ*B (I*κ*B*α*), Phospho(Tyr)-Insulin Receptor Substrate- (IRS-) 1, Phospho(Ser307/312)-IRS1, and Galectin-3 (LGALS3)

Phospho(Ser32)-I*κ*B*α* was measured in the mouse liver and pancreatic tissues and in HepG2 cells by a commercial ELISA (Cell Signaling Technology, Danvers, MA). Sample values were normalized with the total cell protein concentrations, and phospho(Ser32)-I*κ*B*α* was expressed as the percent of the control [2, 3].

Phospho(Tyr)-IRS1 and phospho(Ser307/312)-IRS1 were determined by commercial sandwich ELISAs (Cell Signaling) in the mouse liver and pancreatic tissue homogenates and in HepG2 cell lysates of untreated controls or following exposure to carrageenan, high fat, or the combination of carrageenan and high fat and to exogenous insulin [2, 3].

Serum galectin-3 was measured by DuoSet mouse galectin-3 ELISA (R&D Systems, Bio-Techne, Minneapolis, MN), as described previously [31].

### 2.6. Triglyceride, Cholesterol, and Diacylglycerol Assays

Triglyceride and total cholesterol concentrations in the liver and pancreatic homogenates were measured using commercial assays (Wako Diagnostics, Mountain View, CA) [3]. Diacylglycerol (DAG) concentration was determined in tissue homogenates and in HepG2 cell extracts by a competitive enzyme immunoassay, as per directions (Aviva Systems Biology, San Diego, CA).

### 2.7. Determination of Glucose Uptake

Glucose uptake by HepG2 cells was determined by the Glucose Uptake-Glo™ Assay (Promega, Madison, WI) following exposure to exogenous recombinant human galectin-3 (R&D; 25 mg/L × 4 h). Twelve hours before the assay, the growth medium of the cultured HepG2 cells was removed and replaced with 100 *μ*L DMEM with low glucose (Life Technologies, Thermo Fisher, Carlsbad, CA) without serum. Recombinant human galectin-3 was added to selected wells, and the plates were incubated at 37°C in 5% CO_2_. After 4 hours, the galectin-3-containing medium was replaced with 100 *μ*L DMEM high glucose (4.5 g/L; Life Technologies) ± 10 nM insulin without serum and glucose. Plates were incubated for 1 h at 37°C in 5% CO_2_. Finally, the growth medium was removed, 50 *μ*L of 0.5 mM 2-deoxyglucose was added, and plates were incubated for 30 min at 25°C. The reaction was stopped with stop buffer, neutralization buffer was added, and the 2-deoxyglucose-6-phosphate detection reagent was added. The sample was incubated for 1 h, and then, the luminescence was read in the microplate reader (FLUOstar).

### 2.8. Arylsulfatase B Activity Assay

Arylsulfatase B activity (ARSB) was determined in the liver and pancreas tissues from the mice following 50 weeks of treatment. ARSB was measured using the exogenous substrate 4-methylumbelliferyl sulfate, as previously [31]. Activity was expressed as nmol/mg protein/h.

### 2.9. Binding of Galectin-3 with the Insulin Receptor

The liver and pancreatic tissue homogenates were prepared in cold PBS containing 2 mM EDTA and a complete protease inhibitor cocktail (Bio-Rad, Hercules, CA). Homogenates were sonicated and centrifuged at 10,000 g for 10 min at 4°C. The pellet containing tissue debris and nuclear material was discarded. The collected supernatants were then centrifuged at 100,000 g for 1 h at 4°C. The resulting pellets were solubilized in RIPA buffer to obtain the membrane fraction [32]. The membrane fraction was then incubated overnight at 4°C in a microtiter plate precoated with mouse monoclonal antibody to the insulin receptor (Cell Signaling Technologies). At the end of the incubation, wells were washed thoroughly with wash buffer. The insulin receptor in the membrane preparations bound to the coated insulin receptor antibodies. Galectin-3 molecules associated with insulin receptors were detected by adding biotinylated galectin-3 antibodies (R&D). Finally, the bound biotinylated galectin-3 antibodies were quantified by adding streptavidin-HRP and hydrogen peroxide-TMB substrate. The reaction was stopped by 2N H_2_SO_4_, and the color was measured at 450 nm in a plate reader (FLUOstar). The developed color was proportional to the amount of galectin-3 bound to the insulin receptor in the samples.

### 2.10. QPCR for mRNA Expression of Growth Factor Receptor-Bound Protein (GRB) 10, F4/80, and Peroxisome Proliferator-Activated Receptor- (PPAR-) *α*, *β*/*δ*, and *γ* in Mouse Tissues

Total RNA from the liver, fat, and/or pancreatic tissues of C57BL/6J mice, treated with carrageenan, HFD, or carrageenan and HFD, was extracted, and mRNA expression of GRB10, F4/80, TNF-*α*, and PPAR-*α*, *β*/*δ*, and *γ* was determined by QPCR. Primers were selected using Primer3 software [33] and were as follows: Grb10: (L)5′-AGTGTAGCAGACTTCAGTGGC-3′, (R)5′-TCCAAAACAACCCTGAGCTGT-3′; F4/80: (L)5′-GATGCTCTTCCTGATGGTGAG-3′, (R)5′-CTCCAGATAAACCCCGTCTCT-3′; PPAR-*α*: (L)5′-TGCTCACCCAGCATAGAGAGT-3′, (R)5′-TGTGGACCAAGGACAGAGTG-3′; PPAR-*δ*: (L)5′-CACCTCCTGTTCTTGCTGTCT-3′, (R)5′-GCGTAGTGTTTCTTTGGATGG-3′; and PPAR-*γ*: (L)5′-CACTCGCATTCCTTTGACATC-3′, (R)5′-CGCACTTTGGTATTCTTGGAG-3′.

QPCR was performed as previously [4], and cycle thresholds were calculated and compared with values for *β*-actin.

### 2.11. Oil Red O Staining

Eight to10 mm thick frozen sections of the mouse liver from control, carrageenan-treated, HFD-treated, and combined carrageenan- and HFD-treated were fixed in formalin for 5 min and air-dried. Before staining, slides were washed carefully in tap water, rinsed with 60% isopropanol, and then stained with oil red solution (Sigma-Aldrich) for 15 min. After staining, slides were washed with 60% isopropanol and distilled water and mounted with glycerine.

### 2.12. Confocal Microscopy to Detect Activated Macrophages

Hepatic tissues from the control, carrageenan-treated, HFD-treated, and combined carrageenan- and HFD-treated mice were washed once in 1x PBC containing 1 mM calcium chloride (pH 7.4), fixed for 1.5 hours with 2% paraformaldehyde, and then permeabilized with 0.08% saponin. Preparations were washed with PBS, blocked in 5% normal horse serum (KPL Inc., Gaithersburg, MD), incubated overnight with rabbit anti-mouse monoclonal antibody for F4/80 tagged with green fluorescent dye (Santa Cruz Biotechnology, Dallas, TX) at 4°C, and then washed and exposed for one hour to Alexa Fluor^®^ 488 phalloidin (Invitrogen, Thermo Fisher, Carlsbad, CA) diluted 1 : 40 to stain actin, and slides were coverslipped using DAPI-mounting medium (Vectashield^®^, Vector Laboratories Inc., Burlingame, CA) for nuclear staining. Preparations were washed thoroughly, mounted, and observed using the Zeiss laser scanning confocal microscope LSM710 with ZEN software. The fluorochromes were scanned, and the collected images were exported as czi files for analysis and reproduction.

### 2.13. Statistical Analysis

Data were analyzed using InStat 3 software (GraphPad, La Jolla, CA) and are presented as mean value ± standard deviation (S.D.) of at least three independent biological samples with technical replicates of each determination. The differences between treatments and the control were compared by one-way analysis of variance with the Tukey-Kramer posttest for multiple comparisons, unless stated otherwise. *p* values are represented by ^∗^ or # for *p* ≤ 0.05, ^∗∗^ or ## for *p* ≤ 0.01, and ^∗∗∗^ or ### for *p* < 0.001. Pearson correlation *r* was calculated using Microsoft Excel.

Data are available by communication with the corresponding author.

## 3. Results

### 3.1. Blood Glucose and Insulin Levels, HOMA Scores, and Glucose-Stimulated Insulin Secretion (GSIS) Following Exposure to Carrageenan, High Fat, and Their Combination

**Figure 1:**
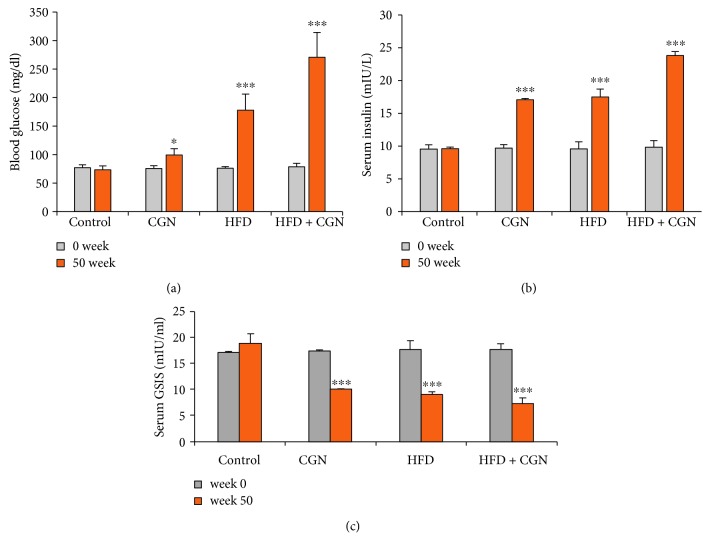
Increases in glucose and insulin follow exposure to carrageenan and HFD. (a) Fasting blood glucose levels increased following carrageenan and high-fat exposures, and the combined exposure following 50 weeks of treatment was significantly great (*p* < 0.001, *n* = 12). Carrageenan exposure had less effect on glucose levels than the HFD. (b) The corresponding fasting insulin levels were increased in the test animals, compared to controls (*p* < 0.001, *n* = 12). (c) The measured glucose-stimulated insulin secretion declined following carrageenan and HFD and by their combination. CGN = carrageenan; GSIS = glucose-stimulated insulin secretion.

Blood glucose and insulin levels were measured in C57Bl/6J mice at the beginning and end of exposure to carrageenan, high fat, or their combination after overnight fasting and 30 minutes after injection of dextrose (2 g/kg IP in filtered PBS) (Figures 1(a) and 1(b)). At the onset, there were no significant differences among the control and experimental groups. Both fasting glucose and insulin levels were significantly (*p* < 0.001) increased at week 50 in all treated groups, as shown in the calculations of the glucose-stimulated insulin secretion (GSIS) (Figure 1(c)) and in the calculated homeostatic assessment of insulin resistance (HOMA-IR), steady state *β*-cell function (%*β*), steady-state % insulin sensitivity (%S), and insulin resistance (IR) scores (Table 1).

HOMA-IR calculation indicated no differences in the HOMA-IR of the different groups at the onset of the intervention (Table 1). After 50 weeks, HOMA-IR increased in the carrageenan-fed mice to ~4-fold the baseline level and to ~7-fold the baseline level in the mice fed the HFD. The combined high fat and carrageenan group showed a synergistic effect, with HOMA-IR increased to ~15 times the baseline.

### 3.2. Increased Phospho(Ser32)-I*κ*B*α* and Phospho(Ser307/312)-IRS-1

**Figure 2:**
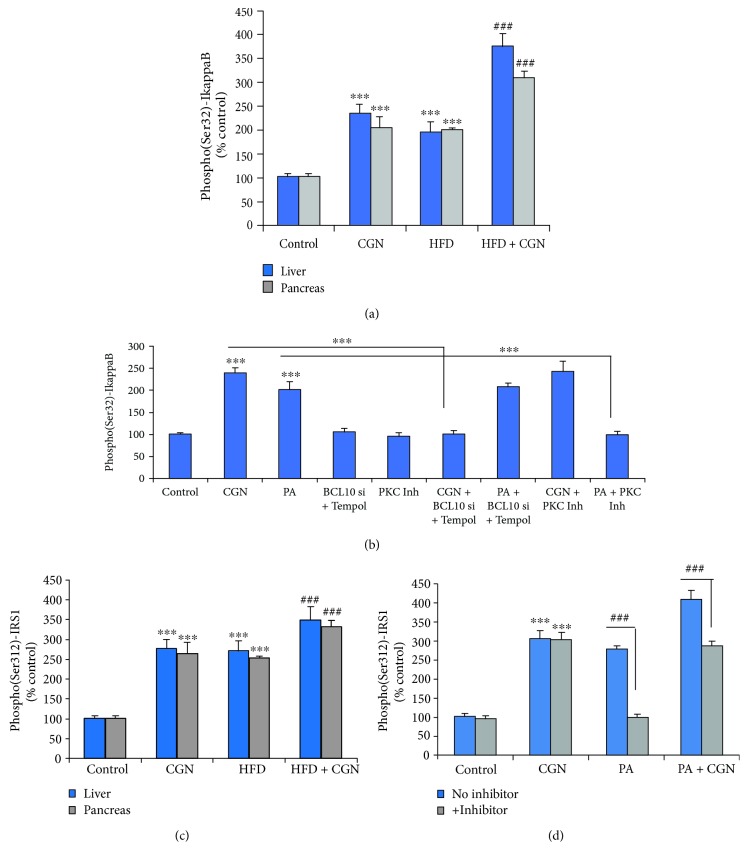
Distinct mechanisms lead to increases in phospho(Ser32)-I*κ*B*α* and phospho(Ser307/312)-IRS1. (a) Phospho-I*κ*B*α* was increased in the hepatic and pancreatic tissues of the mice (*p* < 0.001, *n* = 12). The combination yielded a significantly greater increase (*p* < 0.001). (b) Palmitic acid also increased the phospho(Ser)-I*κ*B*α*. The effect of palmitic acid was inhibited by sotrastaurin but not by the combination of Tempol and BC10 siRNA, which inhibited the carrageenan-induced effects (*p* < 0.001, *n* = 3). (c) Phospho(Ser307/312)-IRS1 increased in the hepatic and pancreatic tissues of the treated animals (*p* < 0.001, *n* = 12). The increase by the combination of carrageenan and HFD was significantly greater. (d) The palmitic acid-induced increase in phospho(Ser307/312)-IRS1 was blocked by exposure to the PKC inhibitor sotrastaurin (*p* < 0.001, *n* = 3). CGN = carrageenan; IRS = insulin receptor substrate; PA = palmitic acid; PKC = protein kinase C.

Carrageenan intake and HFD both significantly increased phospho(Ser32)-I*κ*B*α* (*p* < 0.001) (Figure 2(a)). The combination produced greater increases in both the pancreas and liver tissues. HepG2 cells treated with carrageenan (1 mg/L) or palmitic acid (200 *μ*M) for 24 h also showed significant increases in p-(Ser32)-I*κ*B*α* (Figure 2(b)). When HepG2 cells were treated with 250 nM sotrastaurin, the increase in p-(Ser32)-I*κ*B*α* was completely blocked, with no effect on the carrageenan-induced increase. In contrast, BCL10 siRNA and Tempol (100 nM) × 24 h blocked the effect of carrageenan but not of palmitic acid.

Carrageenan and HFD exposures each significantly increased (*p* < 0.001) the 307/312 phospho(Ser307/312)-IRS-1 in the liver and pancreas, and in combination, the level increased more to ~3.5 times control (Figure 2(c)). When HepG2 cells were treated with carrageenan (1 mg/L) or palmitic acid (200 *μ*M), the phospho(Ser307/312)-IRS-1 increased to ~3 times the baseline, and combined treatment increased the level to ~4 times control (Figure 2(d)). Sotrastaurin (250 nM × 24 h) reversed the effect of palmitic acid but had no impact on the carrageenan-induced increase (Figure 2(d)). These results indicate that the increase in the inflammatory effects by combined carrageenan and HFD was dependent on pathways mediated differentially by PKC or BCL10 and ROS.

### 3.3. HFD Causes the Accumulation of Triglyceride and Increased Diacylglycerol

**Figure 3:**
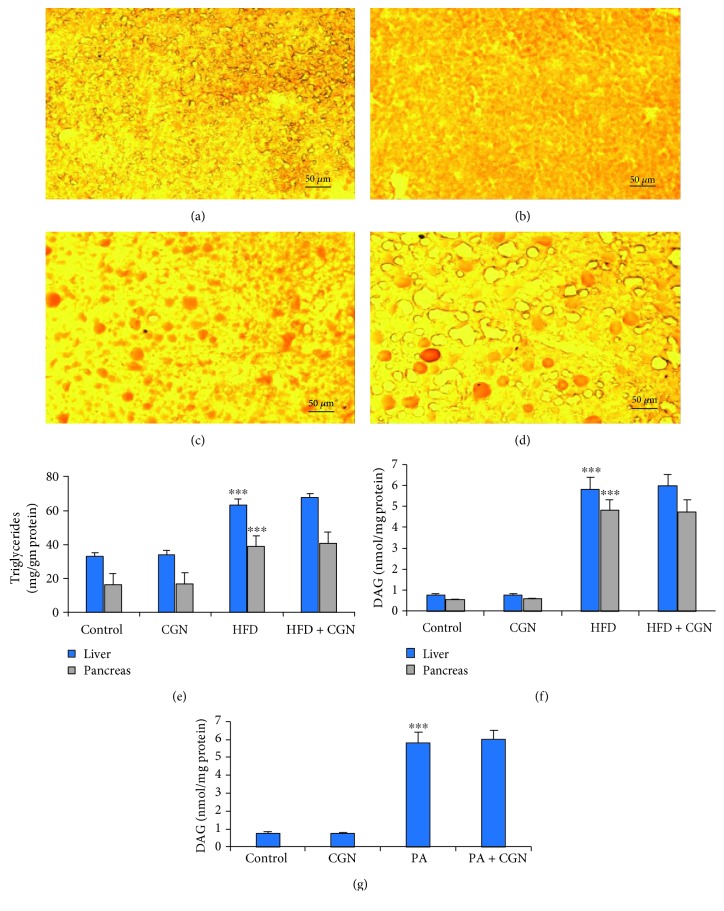
Triglycerides and diacylglycerol are increased by HFD. (a–d) Oil Red O staining showed a marked increase in fat droplets in mouse hepatic tissue following high fat (c) and high fat with carrageenan (d) with a little sign of fat droplets in the carrageenan-treated (b) or control hepatic tissue (a). Scale bar = 50 *μ*m. (e) Triglycerides were measured in the hepatic and pancreatic tissues of the study mice and increased following HFD but not carrageenan (*p* < 0.001, *n* = 12). (f) DAG levels in the hepatic and pancreatic tissues increased following HFD in the mice but not following carrageenan exposure (*p* < 0.001, *n* = 12). (g) Palmitic acid increased DAG in the HepG2 cells (*p* < 0.001, *n* = 3). CGN = carrageenan; DAG = diacylglycerol; PA = palmitic acid.

To further assess how PKC was activated following exposure to high fat, but not by carrageenan, the distinct effects of the exposures were further evaluated. Oil Red O staining of the mouse liver indicated no change compared to the control (Figure 3(a)) following carrageenan (Figure 3(b)). There was a marked increase in the accumulation of fat droplets following the HFD (Figure 3(c)) and to a greater extent by the combination of carrageenan and high fat (Figure 3(d)). The HFD, but not carrageenan, resulted in the accumulation of triglyceride, when measured in the mouse hepatic and pancreatic tissues (Figure 3(e)). The HFD induced the accumulation of diacylglycerol (DAG) to ~6 times the baseline (Figure 3(f)), whereas carrageenan had no effect either alone or in combination with HFD. Palmitic acid (200 *μ*M × 24 h) treatment of HepG2 cells increased the DAG concentration from 0.6 *μ*mol/g protein to 4.4 *μ*mol/g protein (Figure 3(g)), and carrageenan had no effect. Since increase in PKC follows increase in DAG [25], the activation of this pathway by fat, but not by carrageenan, indicates a distinct effect of the HFD.

### 3.4. Increased Macrophage Activation Following Carrageenan or High-Fat Consumption

**Figure 4:**
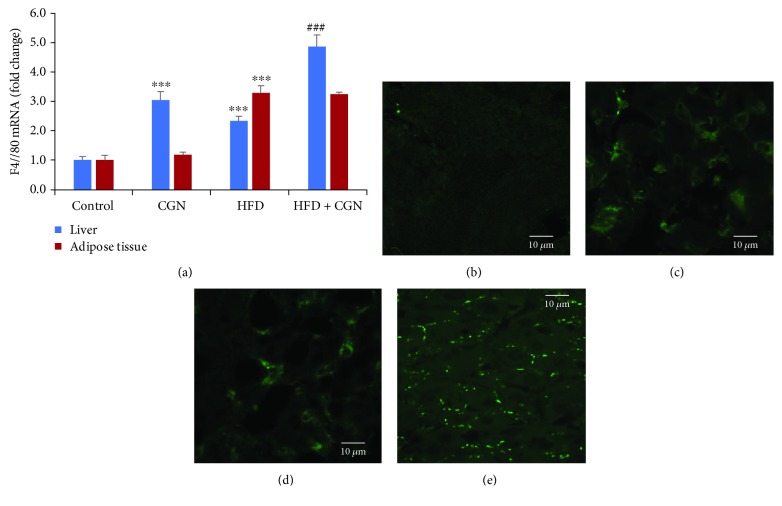
Macrophage activation in the liver and adipose tissue following carrageenan and high-fat exposures. (a) The mRNA expression of F4/80 in the hepatic tissue of the mice showed marked increases in this marker of activated macrophages following carrageenan or HFD and a greater increase following their combination (*p* < 0.001, *n* = 12). In contrast, there was no increase in F4/80 expression in the adipose tissue following carrageenan exposure. (b–e) Confocal images show increase in the F4/80-labeled macrophages following carrageenan or HFD vs. control (green staining). The combination of CGN and HFD markedly increased the F4/80 immunostaining of the activated macrophages. Scale bar = 10 *μ*m. CGN = carrageenan.

Both carrageenan and high fat have been reported to cause macrophage activation [1, 20], leading to inflammation and insulin resistance. The effects of carrageenan and high fat on macrophage activation were evaluated in the hepatic and adipose tissues of the experimental mice. The mRNA expression of the macrophage activation marker F4/80 increased to 3.06 ± 0.28 times the baseline in the hepatic tissue following carrageenan, to 2.34 ± 0.15 times the baseline following HFD, and to 4.87 ± 0.39 times the baseline in combination (Figure 4(a)). In contrast, in the adipose tissue following carrageenan, there was no increase, whereas the expression was 3.29 ± 0.25 times the baseline following high fat. These effects suggest greater effect on macrophage activation by carrageenan in the liver and by high fat in the adipose tissue.

Confocal microscopy to detect activated macrophages by immunostaining for F4/80 was performed in the mouse hepatic tissue. Green fluorescent immunostaining for F4/80 confirmed the increase in activated macrophages following the exposures to carrageenan (Figure 4(c)) or to high fat (Figure 4(d)) compared to the control (Figure 4(b)). Marked increase in the cell surface localization of F4/80 was evident following the combined exposure (Figure 4(e)).

### 3.5. Contribution of Increased Serum Galectin-3 to Insulin Resistance Following Carrageenan or High-Fat Diet

**Figure 5:**
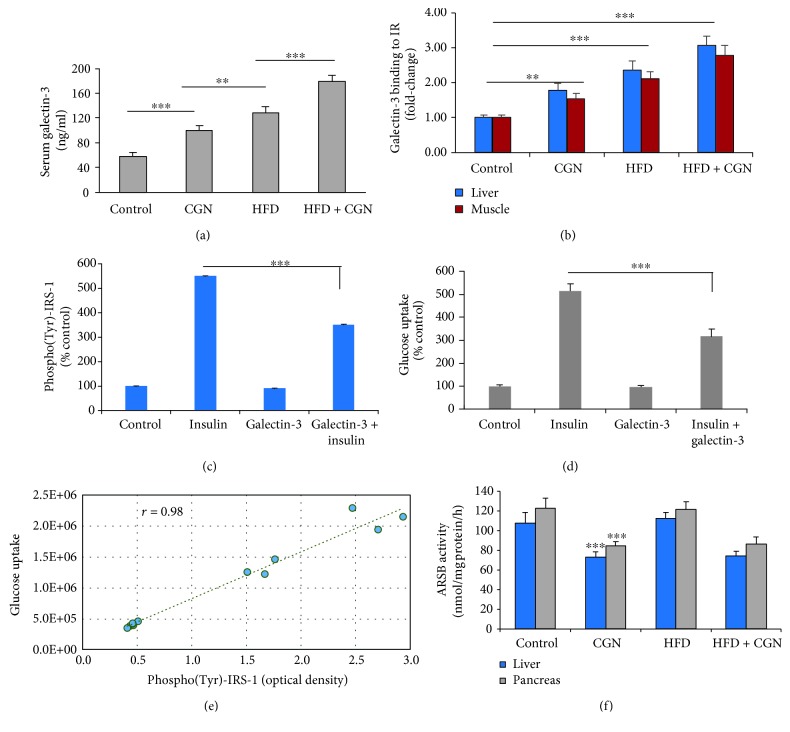
Serum galectin-3 increased following carrageenan or HFD. (a) Serum galectin-3 increased following carrageenan or HFD or their combination (*p* < 0.001, *n* = 18). (b) Galectin-3 binding with the insulin receptor increased in the liver and muscle membrane preparations of the treated mice, with the greatest effect following the combined exposure (*p* < 0.001, *n* = 12). (c) In HepG2 cells, the insulin-induced increase in phospho(Tyr)-IRS1 was significantly inhibited by administration of exogenous recombinant human galectin-3 (*p* < 0.001, *n* = 3). (d) The insulin-induced glucose uptake in the HepG2 cells was blocked by administration of exogenous recombinant human galectin-3 (*p* < 0.001, *n* = 3). (e) The Pearson correlation *r* between the glucose uptake and the phospho(Tyr)-IRS-1 in the HepG2 cells was 0.985. (f) Activity of the enzyme arylsulfatase B (ARSB; N-acetylgalactosamine-4-sulfatase), which removes 4-sulfate groups from the nonreducing end of chondroitin 4-sulfate and dermatan sulfate, was inhibited by exposure to carrageenan, but not by the HFD, in the liver and pancreas of the treated mice (*p* < 0.001, *n* = 12). ARSB = arylsulfatase B; CGN = carrageenan; IRS = insulin receptor substrate.

Macrophage activation is reported to lead to increased serum levels of the *β*-galactoside-binding protein galectin-3 [34], suggesting that serum galectin-3 levels might be increased in the carrageenan- and HFD-treated mice. The mean galectin-3 level increased from 58.2 *μ*g/L to 100.3 *μ*g/L following carrageenan and to 128.3 *μ*g/L following HFD (Figure 5(a)). The serum galectin-3 following combined exposure was 179 *μ*g/L, suggesting an additive effect.

Galectin-3 binding to the insulin receptor (IR) leads to insulin resistance by inhibition of downstream signaling [35]. To consider this mechanism of insulin resistance in the carrageenan and high-fat models, galectin-3-IR binding was measured in hepatic and muscle membrane preparations and shown to increase significantly (*p* < 0.001) (Figure 5(b)). Treatment of HepG2 cells with exogenous recombinant human galectin-3 (25 *μ*g/L × 24 h) significantly inhibited the insulin-induced phospho(Tyr)-IRS-1 and the insulin-stimulated glucose uptake. In HepG2 cells, phospho(Tyr)-IRS-1 increased to ~5 times the baseline, when the cells were challenged with 10 nM recombinant human insulin (Figure 5(c)). Exogenous galectin-3 inhibited this increase (*p* < 0.001) and the insulin-stimulated glucose uptake (*p* < 0.001) (Figure 5(d)). The reduction of tyrosine phosphorylation of IRS-1 was tightly correlated (*r* = 0.985) with the decrease in glucose uptake (Figure 5(e)).

activated macrophages, another possible source of increased galectin-3 is from chondroitin 4-sulfate (C4S), which has *β*-1,3 and *β*-1,4 disaccharide bonds. The increase in serum galectin-3 in the mice may be attributable in part to inhibition of arylsulfatase B (N-acetylgalactosamine-4-sulfatase, ARSB) by carrageenan [36]. ARSB is the enzyme that removes 4-sulfate groups from the nonreducing end of C4S, and galectin-3 binds less with C4S when ARSB is reduced, which may lead to the increased availability of galectin-3 to bind with the insulin receptor [31, 37]. Carrageenan exposure reduced the activity of ARSB in the liver and adipose tissue, whereas HFD had no effect on ARSB activity (Figure 5(f)).

### 3.6. Expression of Peroxisome Proliferator-Activated Receptor- (PPAR-) *α*, *β*/*δ*, and *γ* and of Growth Factor Receptor-Bound Protein (GRB) 10

**Figure 6:**
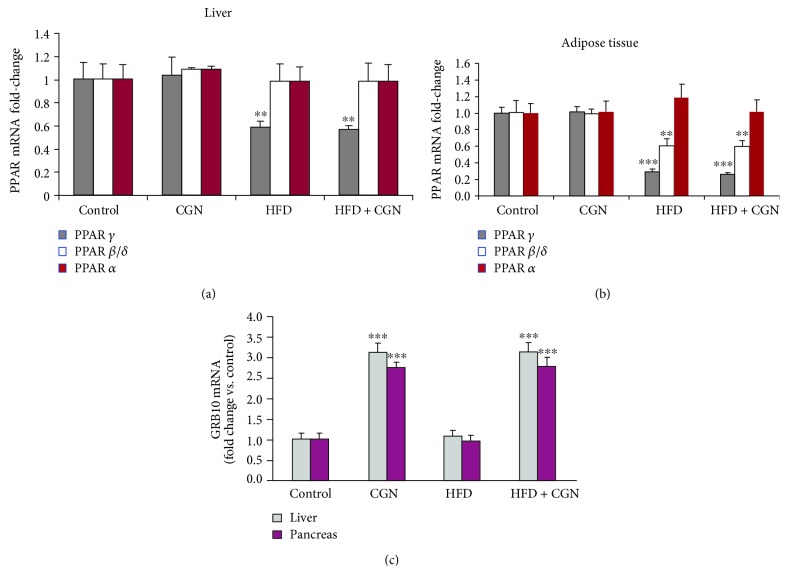
PPAR-*γ* and PPAR-*β*/*δ* are reduced by the HFD; GRB10 is increased by carrageenan and unaffected by high fat. (a) In the mouse hepatic tissue, PPAR-*γ* was significantly reduced by high fat but not by carrageenan (*p* < 0.01, *n* = 12). (b) In the mouse adipose tissue, PPAR-*γ* (*p* < 0.001, *n* = 12) and PPAR-*β*/*δ* (*p* < 0.01, *n* = 12) are significantly reduced following the HFD but not by carrageenan. (c) GRB10 expression was increased in the liver and pancreas of the carrageenan-treated mice but not affected by the HFD (*p* < 0.001, *n* = 12). CGN = carrageenan; GRB = growth factor receptor-bound protein; PPAR = peroxisome proliferator-activated receptor.

mRNA expression of PPAR *α*, *β*/*δ*, and *γ* was determined in the hepatic and adipose tissues of the treated and control mice. HFD, either alone or with carrageenan suppressed PPAR-*γ* (*p* < 0.001) (Figure 6(a)). In hepatic tissue, HFD suppressed PPAR-*β*/*δ*, and carrageenan had no effect (Figure 6(b)). PPAR-*α* did not change significantly following HFD or carrageenan. In the liver and pancreas, GRB10 mRNA expression was significantly increased following carrageenan (*p* < 0.001) but not by HFD (Figure 6(c)).

### 3.7. Interaction among Multiple Pathways of Insulin Resistance

**Figure 7:**
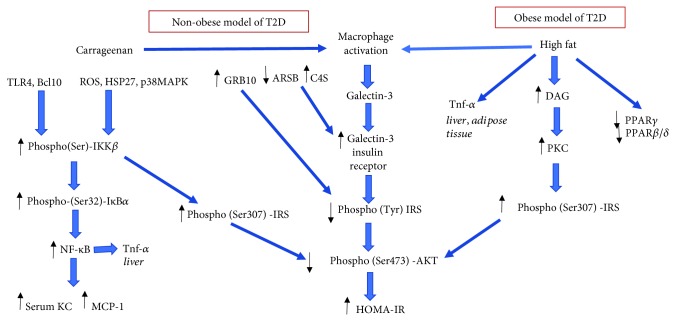
Schematic illustration of pathways by which carrageenan and high fat affect inflammation and insulin signaling. Both carrageenan and HFD lead to activation of inflammatory cascades, leading to increased phospho(Ser)-IRS-1 and decline in phospho(Tyr)-IRS-1. The pathways converge to reduce phospho(Ser473)-Akt and to increase HOMA-IR. Carrageenan has no impact on fat-initiated inflammation in the adipose tissue but has more impact on hepatic inflammation. The two exposures demonstrate the obese and nonobese models of insulin resistance.

The carrageenan model of diabetes is a nonobese model, consistent with the limited impact of carrageenan exposure on triglycerides and on inflammation in the adipose tissue. In contrast, the HFD model is an obese model of diabetes, characterized by the accumulation of triglycerides in the adipose tissue and by activation of macrophages and TNF-*α* in the adipose tissue and liver, although with less impact in the liver than carrageenan. The interacting and unique pathways of carrageenan and high fat in insulin resistance are presented in Figure 7.

## 4. Discussion

This report indicates similar and distinct mechanisms by which carrageenan and high-fat consumption lead to glucose intolerance and insulin resistance. The carrageenan-induced mechanisms involve inflammatory cascades initiated by interaction with TLR4 or by reactive oxygen species. Activation of the IKK signalosome leads to increased phospho(Ser307)IRS-1 and to inhibition of downstream insulin signaling. Carrageenan leads to increased phospho(Ser32)-I*κ*B*α* and nuclear translocation of NF-*κ*B, with enhanced expression of inflammatory mediators, including IL-8 and TNF-*α*. In contrast, HFD increased phospho(Ser307)-IRS-1 and phospho(Ser32)-I*κ*B*α*, but the pathway to these effects involved increases in diacylglycerol (DAG) and protein kinase C (PKC) activity. DAG accumulation has been shown to lead to PKC activation [24, 25] and activated PKC affects phosphorylation of IRS-1 [26].

In our experiments, inhibition of PKC by sotrastaurin blocked the palmitic acid-induced serine phosphorylation of IRS-1 but had no effect on the carrageenan-induced phosphorylation of IRS-1 or I*κ*B*α*. In contrast, the ROS-inhibitor Tempol and BCL10 siRNA blocked the effects of carrageenan, with no impact on the effects of palmitic acid. Hence, both carrageenan and high fat induced the serine phosphorylation of IRS-1 and blocked insulin signaling, but by two distinct pathways.

In addition to serine phosphorylation of IRS-1, both HFD and carrageenan induce TNF-*α* expression. The effect of carrageenan was evident in the liver but not in the adipose tissue, whereas the effect of HFD was greater in the adipose tissue than in the liver. Combined treatment had an additive effect in the liver but not in the adipose tissue. These intersecting and distinct pathways are summarized (Figure 7).

Both carrageenan and HFD lead to an increase in activated macrophages and to increased serum galectin-3 and galectin-3-mediated inhibition of insulin signaling. Carrageenan, unlike HFD, can also increase galectin-3 by effects on arylsulfatase B (ARSB) and chondroitin 4-sulfate (C4S), since carrageenan exposure inhibits ARSB activity [36]. Galectin-3 binds less to C4S when ARSB is reduced [31, 37], potentially contributing to the increase in serum galectin-3 as seen in the experimental mice treated with carrageenan (Figure 5(a)). Also, the increase in C4S when ARSB is reduced has been shown to lead to increased binding of C4S with SHP2, the nonmembrane tyrosine phosphatase (PTPN11). Increased binding of SHP2 with C4S when ARSB is reduced inhibited SHP2 phosphatase activity [38, 39]. This carrageenan-related effect may impact on the tyrosine phosphorylations of the insulin receptor and/or IRS-1 and impair the propagation of insulin signaling, due to sustained tyrosine phosphorylation.

Carrageenan-induced intestinal inflammation is anticipated to provoke the systemic inflammation which leads to insulin resistance. Prior reports have shown the effects of carrageenan intake on the activation of inflammatory cascades in colonic epithelial cells following carrageenan exposure. IL-8 secretion was increased from cultured human colonic epithelial cells by activation of TLR4-BCL10 and by ROS-mediated pathways leading to nuclear translocation of NF-*κ*B and increased expression of IL-8 [5, 6, 11]. In C57BL/6J mice exposed to carrageenan in their water supply, KC, MCP-1, IL-6, and fecal calprotectin were increased [3]. The inflammatory pathways increase phospho-IKK*β*, which interacts with insulin signaling at the level of phospho(Ser307/312)-IRS1 [9]. *In vivo*, carrageenan may exert extraintestinal effects following direct effects on intestinal epithelium, due to changes in the production and secretion of inflammatory mediators, such as IL-8. IL-8 may directly contribute to insulin resistance [40, 41]. Also, carrageenan stimulation of immune cells and influence on the intestinal microbiome might contribute to extraintestinal effects [42–46].

Carrageenan is consumed predominantly as a high molecular weight sulfated polysaccharide which is not expected to be absorbed. However, absorption of carrageenan may occur leading to intracellular inclusions and reduced molecular weight of excreted carrageenan [1, 47, 48]. Carrageenan is composed of sulfated or unsulfated galactose disaccharides linked in alternating *β*-1,4 and *α*-1,3 bonds. The *α*-1,3-galactosidic bonds are not made by enzymes in human cells and are immunogenic [49, 50] and contribute to carrageenan's unique properties.

Dextran sulfate sodium (DSS) is another sulfated polysaccharide which increases intestinal permeability and causes inflammation, but, unlike carrageenan, does not act through TLR4 [51]. A mouse metabolic study comparing the effects of inflammation from HFD with diet-induced obesity and from DSS exposure showed DSS exposure increased cytokines IL-1*β* and IL-12p40 in the liver, mesenteric fat, and subcutaneous fat, without effects on glucose or insulin. In contrast, the HFD increased IL-6 and TNF-*α* in the liver and mesenteric adipose tissue and increased glucose and insulin levels, compared to normal controls [52]. These findings, like those of carrageenan and HFD, indicate that different inflammatory stimuli trigger the activation of different responses which vary in how insulin and glucose metabolism are affected.

Further clarification of the differences between carrageenan and high-fat models of diabetes may help to identify specific metabolic fates associated with activation of innate immunity by carrageenan and with the obese state by high-fat exposure. The current studies provide new insight into how different mechanisms of insulin resistance may be initiated by carrageenan or by high fat, and how several diverse mechanisms may be integrated at the level of IRS-1 phosphorylation.

Reduced dietary intake of carrageenan may lead to improved insulin sensitivity and inhibit development of diabetes.

## Figures and Tables

**Table 1:** HOMA-IR and HOMA 2.2 values.

% (± S.D.)	Control		Carrageenan		HFD		Combined	
	0 wk	50 wk	0 wk	50 wk	0 wk	50 wk	0 wk	50 wk
HOMA-IR	1.76 ± 0.22	1.74 ± 0.20	1.78 ± 0.22	4.19 ± 0.44	1.83 ± 0.15	7.62 ± 0.72	1.87 ± 0.18	15.95 ± 2.92
%*β*	162 ± 25	170 ± 27	164 ± 19	138 ± 17	149 ± 22	50 ± 18	142 ± 22	34 ± 8
%S	82 ± 4	85 ± 4	84 ± 6	45 ± 1	85 ± 8	39 ± 1	84 ± 6	28 ± 10
IR	1.2 ± 0.1	1.2 ± 0.1	1.2 ± 0.1	2.2 ± 0.1	1.2 ± 0.12	2.6 ± 0.1	1.2 ± 0.1	4.4 ± 1.0

## Data Availability

The experimental data used to support the findings of this study are available from the corresponding author upon request.
